# Dynamic Structural Adaptation and Molecular Response of the Digestive Organs During Feed Domestication in *Coilia nasus*

**DOI:** 10.3390/ani16101518

**Published:** 2026-05-15

**Authors:** Aili Sun, Wenxin Hao, Shuangmeng Zhang, Yue Yu, Zexia Gao, Shiming Wan

**Affiliations:** 1 College of Fisheries, Key Lab of Freshwater Animal Breeding, Ministry of Agriculture/Key Lab of Agricultural Animal Genetics, Breeding and Reproduction of Ministry of Education/Engineering Research Center of Green Development for Conventional Aquatic Biological Industry in the Yangtze River Economic Belt, Ministry of Education, Huazhong Agricultural University, Wuhan 430070, China; sunal@webmail.hzau.edu.cn (A.S.); haowenxin@webmail.hzau.edu.cn (W.H.); zsmmmmmm@webmail.hzau.edu.cn (S.Z.); yuyue98983@webmail.hzau.edu.cn (Y.Y.); 2 Hubei Hongshan Laboratory, Wuhan 430070, China

**Keywords:** *Coilia nasus*, feed domestication, organizational structure, enzyme activity, gut microbiota, molecular response

## Abstract

*Coilia nasus*, a nutritionally and economically valuable migratory fish, has long relied on natural bait for its cultivation. However, the inherent instability of natural bait supplies poses a major bottleneck for large-scale aquaculture, necessitating a transition to formulated diets. To elucidate the adaptive response mechanisms underlying this dietary transition, we conducted sampling at three critical stages of feed domestication. This research revealed that while dietary shifts led to moderate alterations in muscle lipid and protein content, overall growth performance remained largely unaffected. During domestication, we observed significant structural remodeling of the stomach, intestine, and liver, along with increased activities of trypsin and lipase. The intestinal microbiota shifted, with Firmicutes abundance increasing notably during the domestication process, although the natural bait group exhibited even higher Firmicutes abundance than the formulated-feed group. Multi-omics analysis of intestinal samples from key domestication stages revealed significant enrichment of lipid metabolism pathways. It suggests that changes in genes and metabolites may be associated with the adaptive response to formulated-feed, including alterations in membrane stability, lipid metabolism and inflammatory state. This study provides the first comprehensive characterization of the structural and molecular adaptations of the digestive system of *C. nasus* during formulated-feed domestication. These findings offer a theoretical foundation for optimizing feeding protocols and supporting the sustainable aquaculture of this species.

## Simple Summary

The *Coilia nasus* is a valuable fish species native to the Yangtze River in China, but its wild populations have declined sharply due to overfishing and habitat loss. Artificial farming has become essential for protecting this species and meeting market demand. However, farmers currently rely on natural bait such as small shrimp and fish to feed these anchovies, which is expensive, unstable in supply, and can introduce diseases. This study explored whether *C. nasus* could be trained to eat formulated feed instead. We examined how its digestive organs, gut microbiota, and metabolic processes changed during the transition from natural bait to formulated feed. The results showed that the fish successfully adapted to formulated feed through structural changes in their stomach and intestines, adjustments in digestive enzymes, and shifts in gut microbiota communities. Importantly, *C. nasus* grew just as well on formulated feed as on natural bait, and their meat even showed improved nutritional quality. These findings provide a scientific basis for farmers to replace natural bait with formulated feed, making anchovy farming more sustainable, cost-effective, and environmentally friendly.

## 1. Introduction

*Coilia nasus*, a nutritionally and economically valuable migratory fish native to the middle-lower Yangtze River and coastal China, has experienced severe population decline since the 1970s due to habitat degradation and overfishing, leading to its inclusion on the IUCN Red List of Threatened Species. Consequently, aquaculture has become essential for conserving and utilizing its germplasm resources. However, the current artificial cultivation of *C. nasus* predominantly relies on natural bait, a practice plagued by high costs, inconsistent supply and quality, poor palatability, and biosecurity risks associated with pathogen transmission [1]. These inherent limitations significantly impede the efficiency and scalability of its aquaculture. Nutritionally balanced and standardized formulated feeds have become the primary feed source for the global aquaculture of economically important fish species, significantly enhancing aquaculture efficiency [2]. Although previous research has found that using light attraction combined with a stepwise transition from slow-sinking to floating pellets can effectively improve the survival rate of *C. nasus* during feed domestication [3], little is known about the changes in digestive organ structure, gut microbiota characteristics, and molecular response signals in the domestication process. This knowledge gap severely restricts the optimization of domestication strategies and the development of the artificial aquaculture industry for *C. nasus*.

The morphological and histological characteristics of fish digestive organs are closely associated with feeding habits, prey size, and dietary diversity [4]. Comparative studies indicate that herbivorous fish exhibit longer and more structurally differentiated intestines than carnivorous species, highlighting a clear relationship between diet and digestive tract structure [5]. Furthermore, different food sources can substantially influence the histological structure of the liver in fish [6]. Therefore, investigating structural differences in digestive organs is essential for understanding fish digestive capacity and elucidating the molecular response mechanisms underlying dietary adaptation. In addition, various digestive enzymes secreted by digestive glands play a critical role in nutrient breakdown and absorption, with their activity levels directly reflecting the organism’s digestive efficiency [7]. Fish digestive enzyme activity is influenced by multiple factors, including intrinsic variables (interspecific and ontogenetic differences) and extrinsic variables (diet and environmental conditions). Among these, dietary composition, serving as the substrate for enzymatic reactions, exerts a pronounced influence on digestive enzyme activity [8]. This relationship has been validated in feed transition studies on Nile tilapia (*Oreochromis niloticus*) [9], blunt snout bream (*Megalobrama amblycephala*) [10], and Pacific white shrimp (*Litopenaeus vannamei*) [11]. Consequently, comparative analysis of digestive enzyme activity across different domestication stages represents a key approach for deciphering the physiological mechanisms of dietary adaptation in fish.

In addition, gut microbiota significantly contributes to nutrient breakdown and absorption in fish. Symbiotic microorganisms in the gut aid in degrading indigestible compounds such as cellulose and chitin, while producing short-chain fatty acids, vitamins, and other bioactive metabolites that enhance host energy utilization [12]. Furthermore, the gut microbiota modulates intestinal permeability and promotes microvilli development, thereby optimizing nutrient absorption [12]. Gut microbiota composition is species-specific and influenced by multiple factors, including environmental conditions, host genetics, and notably, dietary composition [13,14]. Variations in food sources lead to shifts in microbial diversity and functional profile, which subsequently affect nutrient digestion and absorption efficiency [15]. Thus, investigating gut microbiota dynamics during artificial feed domestication is essential to understanding the adaptive mechanisms of fish to dietary transition.

Multi-omics approaches provide novel perspectives and methodologies for elucidating the mechanisms underlying phenotypic formation and their dynamic variations under different conditions. Transcriptomics has been widely applied to the study of mechanisms in fish such as *C. nasus*, including growth [16], gonadal development [17], migration [18], and environmental responses [19]. For instance, a study on Largemouth Bass (*Micropterus salmoides*) revealed that partial replacement of fishmeal with rapeseed meal impaired growth and intestinal integrity via upregulation of the PI3K-Akt and NF-κB pathways [20]. Metabolomics, serving as a bridge between genes and phenotypes [21], has been applied in research on fish nutritional regulation [22], stress responses [23], and physiological characterization [24]. For example, dietary tea polyphenols were shown to elevate amino acid metabolites and enhance related pathways in juvenile hybrid sturgeon (*Acipenser baerii* ♀ × *A. schrenckii* ♂), supporting their role as a natural immunomodulatory feed additive [25]. However, given the complexity of physiological regulation, integrated multi-omics approaches are increasingly employed. For example, in Mandarin Fish (*Siniperca chuatsi*), combined transcriptomic and metabolomic analyses highlighted the PPAR signaling pathway as key in adapting to compound feed, providing new insights into the molecular mechanisms of feed adaptation in it [26].

As feed domestication is essential for establishing artificial culture models, expanding the aquaculture industry, and lowering the threshold for cultivation, understanding how the digestive system responds to dietary change is therefore of particular importance. Specifically, the digestive organs, including the stomach, intestine, and liver, serve as the primary sites where dietary components are processed, nutrients are absorbed, and metabolic responses are initiated. Consequently, investigating structural and molecular alterations in these organs can provide direct insights into the adaptive capacity of fish to dietary changes, making them the most relevant targets for feed adaptation studies. On this basis, this study systematically investigates the structural, digestive, and molecular responses of *C. nasus* during feed domestication. By integrating histology, enzymatic assays, intestinal microbiota profiling, transcriptomics, and metabolomics, we aim to clarify the organismal and molecular adjustments to formulated diet. The findings will support the feasibility of formulated-feed cultivation and provide a theoretical basis for refining domestication strategies, thereby promoting the sustainable development of artificial breeding in this species.

## 2. Materials and Methods

### 2.1. Experimental Materials

**Figure 1:**
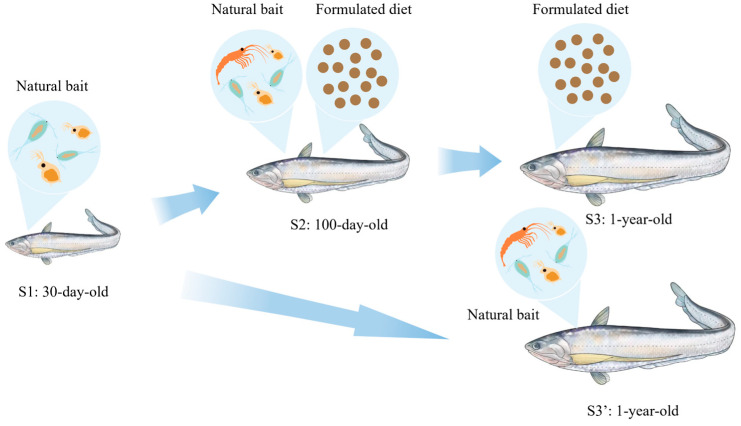
Schematic diagram of the feeding behavior of *C. nasus* at different stages.

Samples of *C. nasus* were collected from Jiangzhiyuan Fisheries Technology Co., Ltd. (Zhenjiang, China), representing four feeding regimes: 30-day-old fish fed exclusively on natural bait (S1); 100-day-old fish in transition receiving mixed natural and formulated diet (S2); 1-year-old fish fully domesticated to formulated diet (S3); and 1-year-old fish maintained solely on natural bait throughout growth (S3′). Natural bait consisted of live rotifers, cladocerans, and copepods, while formulated diet was provided as pelleted diet at scheduled intervals. Feeding behavior across stages is illustrated in Figure 1, and nutritional profiles of both feed types are summarized in Table S1 (3 replicates per feed types and the data expressed as mean ± SEM). To distinguish between developmental and dietary effects, the S3′ group was included as a control. Comparison between S3 and S3′ (same age, different diets) allowed identification of diet-specific changes, while comparison between S1 and S3′ (same diet, different ages) allowed identification of developmental changes. All fish used in this study originated from the same spawning cohort. For each feeding regime, fish were reared in three replicate ponds. Sampling was performed by pooling individuals from the three replicate ponds to minimize pond-specific effects.

### 2.2. Sample Collection and Growth Data Measurement

All fish were anesthetized with 100 mg/L MS-222 before body length (BL) and body weight (BW) were measured for growth analysis (data expressed as mean ± SEM). For each developmental stage, mid-intestinal tissues were sampled as follows: 12 samples were flash-frozen in liquid nitrogen and stored at −80 °C, of which 3 were designated for digestive enzyme assays, 3 for RNA extraction, and 6 for metabolite profiling. Additionally, mid-intestinal contents from 3 samples per stage were frozen for gut microbiota analysis. Tissue samples (intestine, liver, stomach) for histological examination were fixed in 4% paraformaldehyde (3 replicates per tissue per stage). For nutritional analysis, muscle tissues from the S3 and S3′ groups (10 samples per group) were flash-frozen and stored at −80 °C. Pooled samples were used where indicated, with specific masses noted (100 mg for tissue assays, 100 g for muscle composition). All frozen samples were maintained at −80 °C until analysis.

### 2.3. Muscle Nutrient Analysis

Muscle samples from S3 and S3′ stages were analyzed for moisture, ash, crude protein, crude fat, amino acids, and fatty acids. All nutritional indices are presented on a wet weight basis. Muscle samples were directly frozen after collection without lyophilization and were thawed at 4 °C prior to analysis. All data are expressed as mean ± SEM. Moisture was determined by oven drying at 105 °C (GB/T 6435-2014) [27], ash by incineration at 550 °C (GB/T 6438-2007) [28], crude protein by the Kjeldahl method (GB/T 6432-2018) [29], and crude fat by Soxhlet extraction (GB/T 6433-2006) [30]. Amino acids were analyzed using an automatic amino acid analyzer (LA8080, Hitachi, Tokyo, Japan), and fatty acids were quantified with a gas chromatograph (7890A, Agilent, Santa Clara, CA, USA).

### 2.4. Study of the Tissue Structure of Digestive Organs

Stomach, mid-intestine, and liver tissues were dissected from three anesthetized *C. nasus* per stage, rinsed with PBS, and fixed in 5 mL of 4% paraformaldehyde for 24 h at room temperature. Following fixation, tissues were processed through gradient ethanol dehydration, xylene clearing, and paraffin embedding. Serial sections (5 μm thick) were cut, mounted on slides, and subjected to deparaffinization and rehydration. Sections were then stained with hematoxylin (5 min), differentiated with hydrochloric acid–ethanol (3–5 s), blued under running water (10 min), and counterstained with eosin (2 min). After dehydration, clearing in xylene, and sealing with neutral resin, sections were examined, and whole slide images were acquired using a digital slide scanner (LG-FS80, Servicebio, Wuhan, China). The scanned images were analyzed using Slide Viewer 2.5 software. Histological observations were performed qualitatively. For each tissue sample, three non-consecutive sections were examined under a light microscope. Representative images were captured to illustrate the observed structural characteristics, including hepatocyte density, vacuolation, gastric gland cell density, gastric pit morphology, intestinal villus height and density, and goblet cell vacuolation. The descriptions of structural changes were based on comparison of multiple sections from three biological replicates per group. To minimize observer bias, all histological sections were coded with random numbers before evaluation, and the observer was blinded to the sample group identity. All observations were independently reviewed by two researchers. Any discrepancies between the two observers were resolved through discussion.

### 2.5. Digestive Enzyme Activity Analysis

Digestive enzyme activities (α-amylase, lipase, and trypsin) were assayed in mid-intestinal samples from all four developmental stages (S1, S2, S3, and S3′), with three biological replicates per stage (12 samples total). Samples stored at −80 °C were thawed on ice, homogenized in ice-cold 0.8% NaCl (1:4, *w*/*v*), and centrifuged at 4 °C and 3000 r/min for 20 min (3K15, Sigma, Shanghai, China) to obtain the crude enzyme extract. The activities of α-amylase, trypsin, and lipase in these samples were determined using commercial kits from Beyotime Biotechnology (Shanghai, China). Specifically, α-amylase activity was measured using an α-Amylase Activity Colorimetric Assay Kit (Cat# P0405S), trypsin activity was measured using a Colorimetric Trypsin Activity Assay Kit (Cat# P0324S), and lipase activity was measured using an Amplex Red Lipase Activity Assay Kit (Cat# P2777S). All procedures were performed strictly according to the manufacturer’s instructions. Enzyme activities were measured using a microplate reader (Varioskan, Thermo, Waltham, MA, USA) and a UV spectrophotometer (UV-2450, Baizhe, Shanghai, China), with one unit (U) of trypsin activity defined as a ΔA of 0.003 per minute per mg protein at pH 8.0 and 37 °C, one U of lipase as the consumption of 1 μmol substrate per minute per g tissue protein at 37 °C, and one U of α-amylase as the amount hydrolyzing 10 mg starch in 30 min per mg tissue protein at 37 °C.

### 2.6. Characterization of Gut Microbiota Composition Analysis

Mid-intestinal content samples from four developmental stages (S1, S2, S3, S3′; three replicates per stage, 12 samples total) were collected for 16S rDNA-based microbiota analysis. Genomic DNA was extracted using the QIAamp DNA Stool Mini Kit (#51504, Noble Ryder, Beijing, China). The V3–V4 region of the 16S rDNA was amplified using primers 338F (5′-ACTCCTACGGGAGGCAGCAG-3′) and 806R (5′-GGACTACHVGGGTWTCTAAT-3′). Raw paired-end reads were quality-filtered using fastp (v0.22.0, https://github.com/OpenGene/fastp, accessed on 18 March 2024) to obtain high-quality reads. Chimera detection was performed by aligning sequences against a reference database using vsearch (v2.22.1, https://github.com/torognes/vsearch/, accessed on 20 March 2024), and chimeric sequences were removed to generate effective tags. Denoising of the quality-controlled sequences was carried out using the DADA2 plugin in QIIME 2 (v2023.2) to obtain amplicon sequence variants (ASVs). Taxonomic annotation was performed using Mothur (v1.48) against the SILVA 138.1 SSU rRNA database (http://www.arb-silva.de/, accessed on 20 March 2024), with a classification confidence threshold set between 0.8 and 1.0. Based on the rarefied ASV abundance table, alpha diversity indices, including the Chao1 and Shannon indices, were calculated using QIIME 2 (v2023.2). Beta diversity analysis was conducted using the phyloseq package (v1.40.0) in R software (v4.2.0) where weighted UniFrac distances were computed, and principal coordinate analysis (PCoA) was performed to visualize differences in microbial community structure among samples.

### 2.7. Metabolomics Analysis

Mid-intestinal samples from four developmental stages (S1, S2, S3, S3′; six replicates per stage, 24 total) were prepared and analyzed using LC-MS/MS for metabolomic profiling. Raw data acquired via UPLC-MS/MS were processed using Analyst (v1.6.3). Peak extraction, integration, and calibration were performed using MultiQuant (v3.0.3). The preprocessed data matrix was then imported into SIMCA-P (v14.1) for multivariate statistical analysis. Unsupervised principal component analysis (PCA) was first conducted to obtain an overview of overall metabolic differences among groups and the degree of variation within each group. Subsequently, OPLS-DA was performed to maximize separation between groups and identify differential metabolites. Model performance was evaluated using R^2^X and R^2^Y (interpretation ability) and Q^2^ (prediction ability). To prevent overfitting, the model was validated by 200 random permutation tests. Differential metabolites were identified based on variable importance in projection (VIP) values derived from the OPLS-DA model, combined with Student’s *t*-test *p*-values and fold change (FC). To identify key metabolites associated with feed domestication, metabolites exhibiting the same directional changes in both S2/S1 and S3/S2 comparisons and showing significant differences between S3 and S1 were selected. The screening criteria were set as |VIP| > 1, |log_2_FC| > 1, and *p* < 0.05. Concurrently, differential metabolites between S1 and S3′ were identified to capture molecular variations primarily associated with growth and development. Metabolites common to both comparisons were subsequently excluded to filter out background signals related to developmental processes, thereby isolating changes specifically attributable to dietary transition. The identified differential metabolites were imported into the MetaboAnalyst 6.0 online platform (https://www.metaboanalyst.ca/, accessed on 23 March 2024) for metabolic pathway enrichment analysis. Annotation was performed using the KEGG (Kyoto Encyclopedia of Genes and Genomes) database (https://www.kegg.jp, accessed on 15 April 2024) with the hypergeometric test. Pathways with *p* < 0.05 were considered significantly enriched. Enrichment results are presented as bubble plots to visualize key biological pathway changes.

### 2.8. Transcriptomics Analysis

Mid-intestinal tissues from four stages (S1, S2, S3, S3′; three biological replicates per stage) were collected. Total RNA was extracted using Trizol reagent (Cat# 15596026CN, Thermo Fisher, Waltham, MA, USA) following the manufacturer’s protocol. RNA integrity was assessed using a bioanalyzer (2100, Agilent, Santa Clara, CA, USA), and samples with an RNA integrity number (RIN) > 7.0 were used for library construction. Sequencing libraries were prepared using poly(A) mRNA enrichment, fragmented, and reverse-transcribed into cDNA. Libraries were sequenced on the platform (HiSeq 4000, Illumina, San Diego, CA, USA). Sequencing depth was ≥6 Gb of clean data per sample. Raw image data were converted to raw reads (fastq format) by CASAVA. Quality control was performed using fastp (v0.22.0) with the following criteria: removal of adapter-contaminated reads, reads with >10% N content, and reads with >50% low-quality bases (Q ≤ 20). All subsequent analyses were based on the resulting clean data. Clean reads were aligned to the *C. nasus* reference genome (ASM2747535v1, NCBI accession GCA_027475355.1) using HISAT2 (v2.2.1). Gene expression levels were quantified as FPKM (Fragments Per Kilobase of transcript per Million fragments mapped) using FeatureCounts (v2.0.1). Differential expression analysis was performed using DESeq2 (v1.30.1) with Benjamini–Hochberg false discovery rate (FDR) correction. The screening strategy for differentially expressed genes was consistent with that used for differential metabolites as described in Section 2.7. Genes with |log_2_FC| > 1 and *p*adj < 0.05 were considered differentially expressed. KEGG pathway enrichment analysis was conducted using clusterProfiler (v3.18.1), with significance set at *p* < 0.05.

### 2.9. Validation of RNA-Seq Data by Real-Time Fluorescence Quantitative PCR

To validate RNA-seq results, 8 highly expressed mid-intestinal differentially expressed genes (DEGs) were selected for qRT-PCR verification. Primer sequences are listed in Table 1. All primer pairs showed amplification efficiencies within the acceptable range of 90–110%, with R^2^ values ≥ 0.99 (Table S2). Reactions were performed in triplicate using Hieff^®^ qPCR SYBR Green Master Mix (#11201ES03, Yeasen, Shanghai, China) on an instrument (q225 Plus-400, Kubotech, Shanghai, China), with *C. nasus β-actin* as the internal control. The thermal cycling protocol included an initial denaturation at 95 °C for 30 s, followed by 40 cycles of 95 °C for 5 s, 60 °C for 30 s, and 72 °C for 30 s. Relative gene expression was calculated using the 2^−ΔΔCt^ method, and statistical significance (*p* < 0.05) was assessed by *t*-test in GraphPad Prism 8.0.2.

### 2.10. Statistical Analysis

All data are presented as mean ± standard error of the mean (SEM). Normality of the data was assessed using the Shapiro–Wilk test, and homogeneity of variances was tested using Levene’s test. For comparisons between two groups, the independent samples *t*-test was performed. For comparisons among three or more groups, one-way analysis of variance (ANOVA) was used, followed by Tukey’s HSD post hoc test for multiple comparisons. The significance level was set at *p* < 0.05 for all analyses. All statistical analyses were conducted using GraphPad Prism 8.0.2 software.

## 3. Results

### 3.1. Growth Performance in C. nasus Is Not Markedly Altered by Feed Type

**Figure 2:**
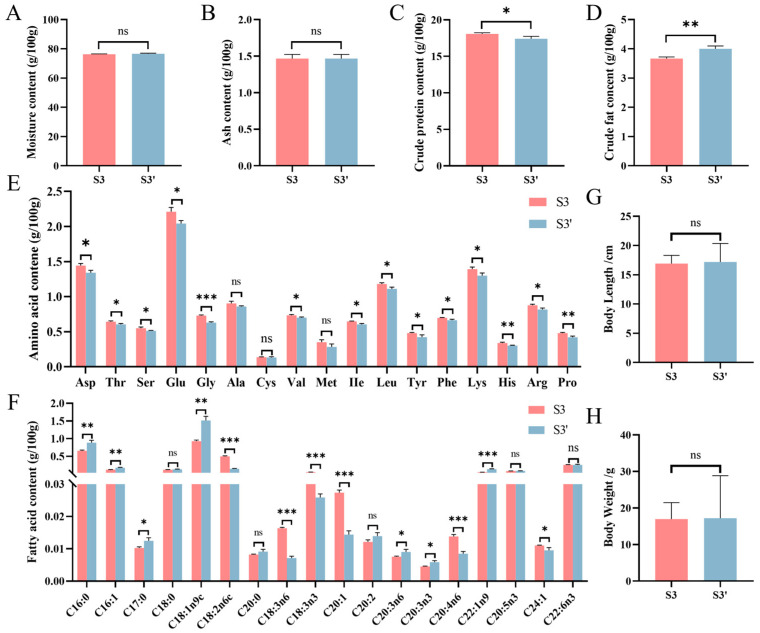
Evaluation of growth performance and nutrient composition of *C. nasus* at different stages. (**A**–**D**) Comparison of moisture, ash, crude protein, crude fat content between the S3 and S3′ groups. (**E**,**F**) Comparison of amino acid and fatty acid content. (**G**,**H**) Comparison of body length and body weight between two groups. Red and blue bar denote the S3 and S3′ stages, respectively. All data are presented as mean ± SEM (*n* = 3 for nutrient analyses; *n* = 70 for growth measurements) “ns”, no significance; “*”, *p* < 0.05; “**”, *p* < 0.01; “***”, *p* < 0.001.

To assess the feasibility of replacing natural bait with formulated diet in *C. nasus* aquaculture, we compared growth parameters and muscle nutrient profiles between 1-year-old fish fed formulated diet (S3) and those maintained on natural bait (S3′). No significant differences (*p* > 0.05) were observed in muscle moisture or ash content between the two groups (Figure 2A,B). However, protein content was significantly higher in the S3 group (*p* < 0.05; Figure 2C), whereas lipid content was markedly elevated in the S3′ group (*p* < 0.01; Figure 2D). Furthermore, the muscle amino acid profile was substantially influenced by feed type. Most essential amino acids (e.g., threonine, valine, isoleucine, leucine, lysine) as well as flavor amino acids (e.g., glutamic acid, aspartic acid, glycine, arginine) were present at significantly higher levels in the S3 group (Figure 2E). Of the 18 fatty acids detected in muscle, 13 showed significant differences between the S3 and S3′ groups (Figure 2F). The contents of both essential fatty acids (e.g., C18:2n6c, C18:3n3) and the semi-essential fatty acid C20:4n6 were higher in the S3 group. Moreover, the total content of unsaturated fatty acids in the muscle of *C. nasus* from the S3 group (0.6393 ± 0.01339 g/100 g) was significantly greater than that in the S3′ group (0.3335 ± 0.01461 g/100 g; Table S3). However, although the muscle nutrient profiles differed between two groups, no significant growth variation in body length or weight was observed (Figure 2G,H). These findings indicate that formulated diet can sufficiently fulfill the nutritional needs of *C. nasus* during feed domestication and effectively sustain its growth.

### 3.2. The Feed Domestication of C. nasus Is Accompanied by Significant Histological Alterations in the Digestive Organs

**Figure 3:**
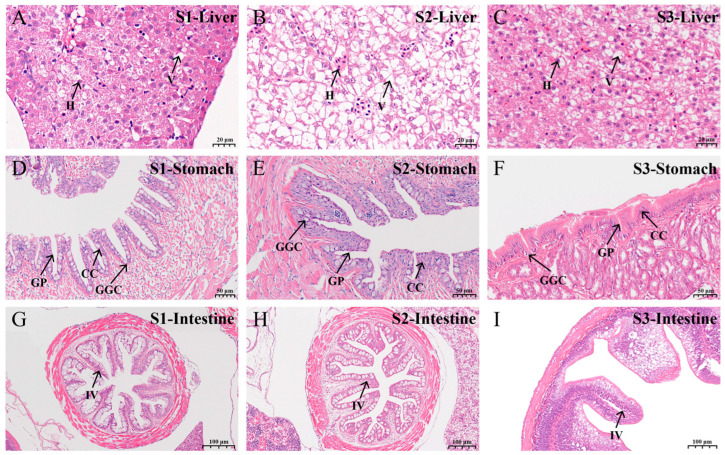
Structural characteristics of the digestive organs during feed domestication of *C. nasus*. (**A**–**C**) Hepatic tissue structure from S1 to S3. (**D**–**F**) Gastric tissue structure from S1 to S3. (**G**–**I**) Intestinal tissue structure from S1 to S3. Scale bars are shown in the lower right corner. H, hepatocyte; V, Vacuole; GGC, gastric gland cells; GP, gastric pits; CC, columnar cells; IV, intestinal villus.

The organizational structure observation at 3 key stages of feed domestication (S1, S2, S3) revealed pronounced morphological changes in the liver, stomach and intestine. The hepatocyte density appeared to decline followed by recovery, while the hepatocellular vacuolation rate showed an increasing trend at stage S2 before subsequently decreasing (Figure 3A–C). Comparative analysis of gastric tissues across the 3 stages revealed a progressive increase in gastric gland cells. In stage S3, a distinct muscular layer of the gastric glands had formed, accompanied by an increased gastric pits number, greater tissue loosening, and significant elongation of columnar cells (Figure 3D–F). Similarly, observation of intestinal morphology across stages demonstrated apparent increases in intestinal diameter, height of mucosal folds, and villus density (Figure 3G–I). These results indicate that during the initial phase of dietary transition, the formulated feed induced physiological stress on the digestive organs of *C. nasus*. However, prolonged acclimation promoted adaptive modification of the organizational structure, which enhanced the digestive and absorption capacity for the formula feed, thereby mitigating the adverse effects caused by the bait change.

**Figure 4:**
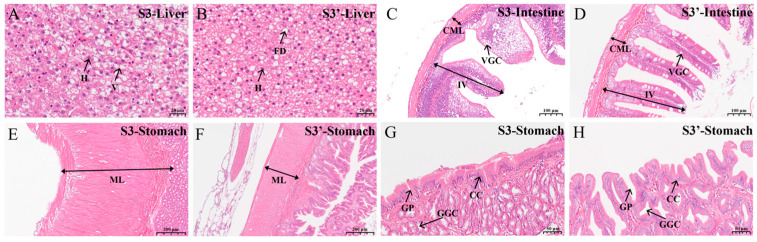
Characteristics of digestive organs in S3 vs. S3′ comparison group. (**A**) Liver tissue in stage S3. (**B**) Liver tissue in stage S3′. (**C**) Intestine tissue in stage S3. (**D**) Intestine tissue in stage S3′. (**E**,**G**) Gastric tissue in stage S3. (**F**,**H**) Gastric tissue in stage S3′. Scale bars are shown in the lower right corner. H, hepatocyte; V, vacuole; FD, fat droplets; IV, intestinal villus; CML, circular muscle layer; VGC, vacuolated goblet cells; ML, muscular layer of stomach wall; GGC, gastric gland cells; GP, gastric pits; CC, columnar cells.

Furthermore, significant morphological differences were also observed in the digestive organs between the S3 group (fed formulated feed) and S3′ group (fed natural bait), both with established feeding habits. Comparative analysis revealed that the S3′ group exhibited higher hepatocyte density, smaller nuclear size, more lipid droplets, and a lower vacuolation rate than the S3 group (Figure 4A,B). In intestinal structure, although the intestinal diameter showed little variation between the two groups, the S3′ group demonstrated greater height of mucosal folds, thicker circular muscle layer, and higher villus density compared to the S3 group, whereas vacuolation of goblet cells was more pronounced in the S3 group (Figure 4C,D). Comparative analysis also indicated that the S3′ group had a relatively thinner muscular layer of the gastric wall and lower gastric gland cell density. The gastric pits in the S3′ group displayed higher tissue loosening, but their number showed no significant difference compared to the S3 group. Additionally, while columnar cells were longer in the S3′ group, their density remained similar between the two groups (Figure 4E–H). These findings further demonstrate that dietary modification induces adaptive structural changes in the digestive organs of *C. nasus*, thereby supporting its gradual physiological adaptation to feed domestication.

### 3.3. Intestinal Trypsin and Lipase Activities Increase Significantly During the Feed Domestication of C. nasus

**Figure 5:**
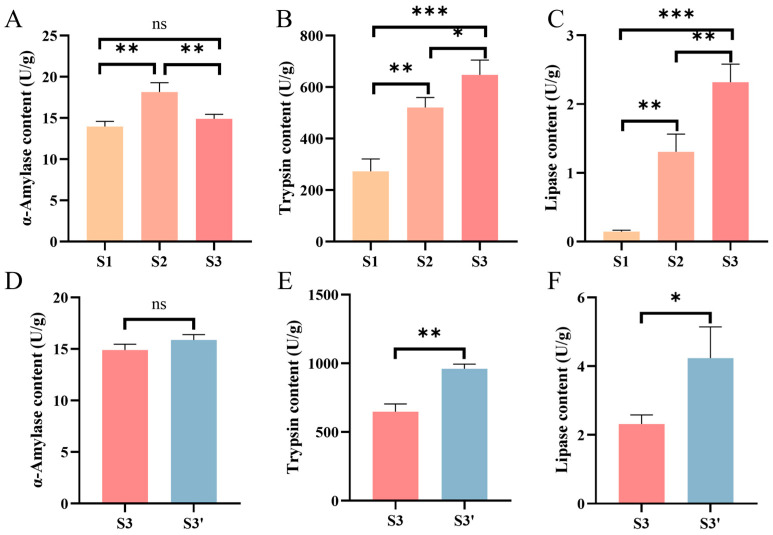
Evaluation of intestinal digestive enzyme activities at different feed domestication stages in *C. nasus*. (**A**–**C**) Comparison of intestinal α-amylase, trypsin, and lipase activities among the S1, S2, and S3 groups. (**D**–**F**) Comparison of intestinal α-amylase, trypsin, and lipase activities between the S3 and S3′ groups. Yellow, orange, red, and blue bar denote the S1, S2, S3, and S3′ stages, respectively. All data are presented as mean ± SEM (*n* = 3 per group) “ns”, no significant difference; “*”, *p* < 0.05. “**”, *p* < 0.01, “***”, *p* < 0.001.

To characterize changes in digestive capacity during the feed domestication of *C. nasus*, intestinal digestive enzyme activities (α-amylase, lipase, and trypsin) were measured at 3 key domestication stages. The results showed that α-amylase activity initially increased and then decreased across the S1 to S3 stages (*p* < 0.05, Figure 5A), whereas trypsin and lipase activities exhibited a gradual upward trend (*p* < 0.05, Figure 5B,C). In addition, intestinal digestive enzyme activities were compared between 1-year-old fish fed different diets. Trypsin and lipase activities were significantly lower in the S3 group (formulated feed) than those in the S3′ group (natural bait) (*p* < 0.05, Figure 5E,F). These findings reflect the differences in digestive enzyme requirements for different feed types, indicating that the intestinal digestive capacity of *C. nasus* undergoes dynamic adjustments in response to dietary changes during feed domestication, enabling efficient breakdown and utilization of different feed types.

### 3.4. Dynamic Changes in Gut Microbiota Promote Nutrient Digestion and Absorption During Feed Domestication of C. nasus

**Figure 6:**
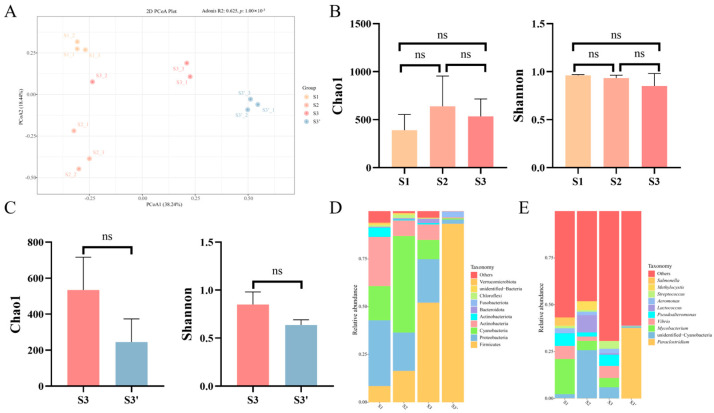
Gut microbiota composition in *C. nasus* under different dietary components. (**A**) Principal coordinate analysis (PCoA) based on weighted UniFrac distances. (**B**) Chao1 and Shannon diversity indices of microbiota across domestication stages. (**C**) Chao1 and Shannon indices of microbiota between different diet groups. (**D**,**E**) Phylum-level (**D**) and genus-level (**E**) relative abundance of the gut microbiota across domestication stages and dietary regimes. Yellow, orange, red, and blue denote the S1, S2, S3, and S3′ stages, respectively. All data are presented as mean ± SEM (*n* = 3 per group) “ns”, no significant difference.

To elucidate the response of gut microbiota composition and diversity to dietary domestication and its impact on intestinal digestive capacity in *C. nasus*, intestinal contents from each stage (S1, S2, S3, and S3′) were subjected to 16S rDNA sequencing. A total of 980,076 valid sequences were obtained, which were clustered at 100% similarity, yielding 3220 amplicon sequence variants (ASVs). The numbers of ASVs detected at S1, S2, S3 and S3′ were 376, 604, 511 and 210, respectively (Table 2). The coverage of the detected gut microbiota was close to 1 at all stages, indicating that the sequencing results reliably reflect the actual microbial community (Table 2). Principal coordinates analysis (PCoA) based on weighted UniFrac distance showed an apparent separation of the microbiota among the four groups. The first two principal components accounted for 38.24% and 18.44% of the variation, respectively, suggesting structural differences in the gut microbiota across these stages (Figure 6A).

We then evaluated the Alpha diversity index of the gut microbiota. The results showed that the Chao1 index exhibited an initial increase followed by a decrease, while the Shannon index showed a slight decline during the domestication of *C. nasus*, though neither change was statistically significant (Figure 6B). At the phylum level, comparison among the S1, S2, and S3 groups revealed a significant increase in the abundance of Firmicutes, a decrease in Proteobacteria, and an initial rise followed by a decline in Cyanobacteria (Figure 6D). At the genus level, *Mycobacterium* displayed a decreasing trend, *Lactococcus* and unidentified Cyanobacteria first increased and then decreased, while *Vibrio* showed an initial decrease followed by an increase (Figure 6E). Comparative analysis further indicated that the Chao1 and Shannon index were slightly higher in the S3 group (fed formulated diet) than in the S3′ group (fed natural bait), though the differences were not significant (Figure 6C). Further comparison of relative microbial abundance demonstrated that the proportions of Proteobacteria, Actinobacteria, and Cyanobacteria were significantly higher in the S3 group, whereas Firmicutes was significantly more abundant in the S3′ group at the phylum level (Figure 6D). At the genus level, the composition of the gut microbiota differed markedly between the two groups: *Paraclostridium* dominated in the S3′ group (37.58%) but accounted for only 0.06% in the S3 group. In contrast, unidentified Cyanobacteria, *Mycobacterium*, *Vibrio*, and *Pseudoalteromonas* collectively comprised 23.28% in the S3 group, whereas these four taxa accounted for only 1.02% in the S3′ group (Figure 6E). These findings suggest that both the abundance and diversity of the gut microbiota undergo dynamic changes during the domestication of *C. nasus*, and differences in microbial composition and abundance may indirectly influence the fish’s ability to digest and absorb different nutrients.

### 3.5. Intestinal Multi-Omics Analysis of Tissues Reveals Metabolic and Transcriptional Response Characteristics During Domestication of C. nasus

**Figure 7:**
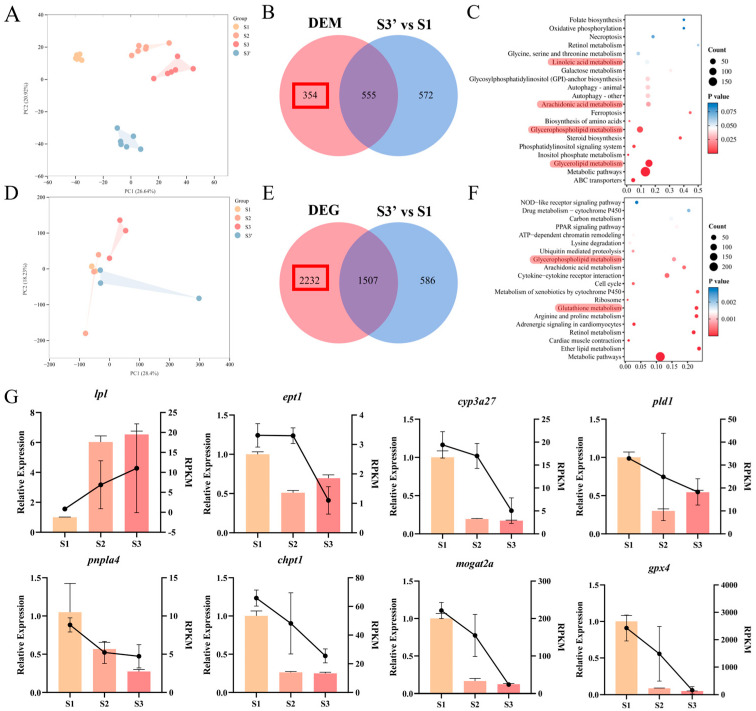
Multi-omics analysis of intestine in *C. nasus* at different domestication stages. (**A**) Principal component analysis of metabolome data at different stages. (**B**,**C**) Screening of differential metabolites and KEGG pathway enrichment analysis. Pathways associated with nutrient digestion and absorption are highlighted in red. (**D**) Principal component analysis of transcriptome data at different stages. (**E**,**F**) Screening of differentially expressed genes and KEGG pathway enrichment analysis. Pathways associated with nutrient digestion and absorption are highlighted in red. (**G**) Validation of RNA-seq gene expression by qRT-PCR. Yellow, orange, red, and blue denote the S1, S2, S3, and S3′ stages, respectively. All data are presented as mean ± SEM (*n* = 6 for metabolomics; *n* = 3 for transcriptomics and qPCR analysis).

Multi-omics analysis offers a robust technology for elucidating metabolic profiles and their underlying molecular regulation during the dietary domestication of *C. nasus*. Metabolomic analysis was first performed to identify differentially accumulated metabolites. Principal component analysis (PCA) of metabolomic data from the 4 groups revealed that principal components 1 and 2 explained 26.64% and 20.92% of total variance, respectively, clearly distinguishing the groups and reflecting substantial metabolic diversity among them (Figure 7A). To exclude developmental effects and focus on diet-responsive molecular changes, we adopted a two-step screening strategy as described in the methods section. Specifically, differential metabolites and genes that appeared in both the S1 vs. S3′ comparison (developmental effect) and the S3 vs. S1 comparison (combined effect) were removed from subsequent analysis, leaving only those specifically associated with dietary transition from natural bait to formulated feed. Finally, a total of 354 differential metabolites belonging to 14 distinct classes were identified ultimately, including phosphatidylcholine (PC), lysophosphatidylcholine (LPC), phosphatidylethanolamine (PE), etc. (Figure 7B and Table S4). KEGG enrichment analysis revealed that these metabolites were significantly enriched in pathways including glycerolipid metabolism, glycerophospholipid metabolism and arachidonic acid metabolism (Figure 7C), indicating their close association with dietary adaptation in *C. nasus* and suggesting their potential as biomarkers for intestinal metabolic adaptation.

Subsequently, transcriptomic analysis was conducted to explore the underlying response mechanisms. PCA of intestinal transcriptome data showed that the first two principal components accounted for 28.4% and 18.23% of variance, respectively, with clear separation among the 4 groups (Figure 7D). Following the same screening strategy described in the methods section, a total of 2232 differentially expressed genes were identified, including *chpt1*, *ept1*, *lpl*, *pla2g4c*, *dgki*, *gpx4*, etc. (Figure 7E and Table S5). These genes were also significantly enriched in pathways such as glycerophospholipid metabolism and arachidonic acid metabolism (Figure 7F).

Moreover, to ensure the accuracy of the expression profiles obtained from RNA-seq, 8 differentially expressed genes were randomly selected for validation by qPCR analysis. The qPCR results showed that the expression trends consistent with those revealed by the transcriptomic analysis, which confirms the reliability of the sequencing data (Figure 7G).

### 3.6. Synergistic Regulation of Lipid Metabolism-Related Genes and Metabolites May Promote Feed Domestication in C. nasus

Pathway enrichment indicates that molecular regulation of lipid metabolism plays a critical role during the domestication of *C. nasus*. To systematically elucidate the response mechanism, we analyzed dynamic changes in gene expression and metabolite synthesis within related signaling pathways. The results showed that although the expression of phospholipid transferases *chpt1* and *ept1* was downregulated during domestication, reduced expression of *pld1* decreased phosphatidic acid (PA) production, thereby inhibiting the consumption of phosphatidylcholine (PC) and phosphatidylethanolamine (PE) and increasing their levels. As major components of the cell membrane, elevated PC and PE may enhance intestinal epithelial membrane stability, adapting to changes in the lipid composition of the feed. At the same time, upregulation of *dgki* promoted the conversion of PA to diacylglycerol (DG), which synergized with DG accumulation resulting from the downregulation of *dgke* and *dgkh*, collectively driving triacylglycerol (TG) synthesis and accumulation. Metabolomic analysis revealed decreased levels of degradation products such as lysophosphatidylcholine (LPC), free fatty acids (FFAs), and PA, while synthetic lipids including PC, PE, and TG increased significantly. This trend indicates a metabolic preference for converting lipids into energy storage forms. Moreover, upregulation of *alox15b* and downregulation of *gpx4* inhibited the production of pro-inflammatory mediators 5-HETE and 15-HETE, maintaining a low-inflammatory state in the intestine. Accumulation of epoxy fatty acids (9,10-EpOME and 12,13-EpOME) enhanced fatty acid transport efficiency and, together with membrane phospholipid saturation, established an antioxidant defense system.

**Figure 8:**
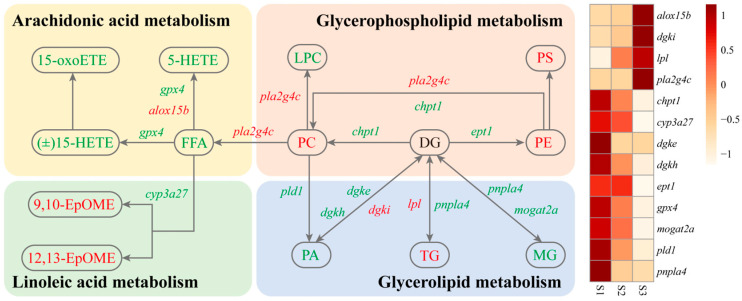
Key molecular regulatory network during feed domestication of *C. nasus*. Red indicates upregulated metabolites or genes, green indicates downregulated metabolites or genes. Darker colors represent higher gene expression levels in the gene expression heatmap.

In summary, through coordinated regulation of gene expression and metabolite levels, *C. nasus* enhanced intestinal lipid digestion and absorption capacity while maintaining membrane stability and energy supply. By modulating arachidonic acid metabolism to sustain intestinal homeostasis, it achieved metabolic adaptation to formulated diet (Figure 8).

## 4. Discussion

The successful artificial culture of *C. nasus* offers a new approach for the conservation and sustainable utilization of this species. However, the traditional practice of natural bait feeding faces challenges such as high costs, inconsistent supply, and disease transmission risks, underscoring the need for feed domestication. Domestication of high-value fish can lower labor and production costs while accelerating the development of aquaculture [31]. Multiple studies have demonstrated that replacing natural bait with formulated feed does not affect the growth performance of aquaculture species, and the results of this study provide further support for this perspective [2,32,33]. Feed type and composition determine muscle quality and nutritional value by influencing the content of amino acids, polyunsaturated fatty acids (PUFAs), and other components [34]. In this study, the muscle of *C. nasus* fed formulated diet showed significantly higher levels of most essential amino acids and PUFAs. This confirms that formulated feed can meet the comprehensive nutritional requirements of *C. nasus*, ensuring its value as a food fish.

A potential concern regarding the experimental design is that dietary transition is confounded with developmental stage, as fish in the S1, S2, and S3 groups differ not only in diet but also in age. To address this issue, the S3′ group (1-year-old fish fed natural bait throughout growth) was included as a critical control. This design allowed us to disentangle the two effects as follows. First, comparison between S3 and S3′ (same age, different diets) specifically reflects diet-induced changes without developmental interference. Second, comparison between S1 and S3′ (same diet, different ages) captures developmental changes independent of diet. Third, in multi-omics analyses, differential metabolites and genes that appeared in both the S1 vs. S3′ comparison (developmental effect) and the S3 vs. S1 comparison (combined effect) were excluded, thereby removing developmental background signals. Using this strategy, we aimed to identify molecular responses specifically attributable to the transition from natural bait to formulated feed. Nevertheless, we acknowledge that a fully crossed experimental design would allow more complete separation of developmental and dietary effects. For example, including a 30-day formulated diet group and additional time points, which should be addressed in future studies. Digestive organ morphology and digestive enzyme activity are fundamental indicators of digestive function in fish and serve as important criteria for evaluating domestication success [35]. In this study, significant adaptive changes occurred in the stomach and intestinal histological structures of *C. nasus* during domestication, suggesting morphological remodeling in response to the formulated feed. As the primary site of lipid metabolism, the liver exhibits histological changes that sensitively reflect physiological disturbances caused by nutritional imbalance or deficiency [36]. After domestication of *C. nasus*, the hepatocyte counts recovered and vacuolation rates decreased markedly. This indicates that during the feed domestication process, the coordinated interplay between individual growth and feeding habit transition in *C. nasus* drives adaptive structural and functional adjustments in the key digestive organs, mitigating the physiological stress of dietary transition. Feed composition also significantly influences digestive enzyme activity [37]. Trypsin and lipase activities were lower in fish fed formulated feed than in those fed natural bait, but both enzymes showed a gradual increasing trend during domestication. This suggests that intestinal digestive capacity undergoes adaptive changes depending on feed type, and is improved during the growth and development of *C. nasus* as well as through adaptation to formulated feed, thereby enabling more efficient digestion and utilization of different diets. Although α-amylase activity did not differ significantly between the S3 and S3′ groups, it exhibited an initial rise followed by a decline during domestication. This pattern may reflect a compensatory response to starvation during the early transition phase, as fish increased carbohydrate metabolism to meet energy demands [38]. Then activity normalized after adaptation, although the underlying mechanisms require further study.

The gut microbiota plays an active role in host digestion and metabolism, and its dynamic community structure reflects physiological adaptation and homeostasis during domestication [39]. Shifts in microbial composition and abundance can indirectly indicate host physiological status and nutrient utilization capacity [40]. In the present study, during the transition from natural bait to formulated diet (S1 to S3), we observed a decrease in the relative abundance of Proteobacteria and an increase in Firmicutes. Proteobacteria has been reported as a potential marker of gut dysbiosis in some fish species [41], and its decline during domestication may suggest good tolerance of *C. nasus* to the formulated diet. However, it should be noted that the natural bait group (S3′) exhibited even lower Proteobacteria abundance than the formulated-feed group (S3), yet both groups showed normal growth performance. This observation indicates that Proteobacteria abundance alone is not a definitive indicator of intestinal health, and its interpretation should be context-dependent. The increase in Firmicutes abundance has been associated with enhanced inflammation resistance and intestinal barrier function in some studies [42]. Within this phylum, *Lactococcus* is considered a promising probiotic candidate in aquaculture [43]. Certain *Lactococcus* species have been reported to improve feed palatability, modulate gut microbiota and microvilli morphology, promote feed intake, and support growth [44]. In our study, Lactococcus abundance initially increased during the early domestication stage (S1 to S2), which may have facilitated the transition to formulated-feed, and then declined after full adaptation (S2 to S3). Furthermore, Paraclostridium, a genus known to produce butyrate [45], was more abundant in the natural bait group (S3′) than in the formulated-feed group (S3). Butyrate has been shown to enhance intestinal digestive capacity in *Pelteobagrus fulvidraco* [46], suggesting that supplementation with butyrate or its salts in formulated diets could be a potential strategy for maintaining intestinal health in *C. nasus*. Nevertheless, these interpretations are based on compositional data and literature correlations; direct functional evidence is needed to confirm the proposed roles of specific taxa in subsequent research. Overall, while the natural bait group showed certain advantages in microbiota composition, our findings demonstrate that *C. nasus* successfully achieved effective utilization of formulated feed after domestication through a combination of structural remodeling of digestive organs, adjustments in digestive enzyme activities, and succession of gut microbial communities. Importantly, no significant differences in growth performance were observed between the S3 and S3′ groups, confirming the feasibility of replacing natural bait with formulated feed from a practical perspective.

Integrated transcriptomic and metabolomic analyses indicate that domestication of *C. nasus* involves specific changes in the expression of genes such as *pld1*, *dgki*, and *alox15b*, along with corresponding shifts in metabolites such as phosphatidylcholine, triacylglycerol, and 5-HETE. It is shown that lipid metabolism-related pathways, including glycerolipid metabolism, glycerophospholipid metabolism, and arachidonic acid metabolism, were significantly enriched during feed domestication in *C. nasus*. Based on these correlative data, we propose the following hypothetical model for the molecular response to formulated diet adaptation. First, the downregulation of *pld1* expression reduces phosphatidic acid (PA) production, which may inhibit the consumption of phosphatidylcholine (PC) and phosphatidylethanolamine (PE), leading to their accumulation. As major components of cell membranes, increased levedls of PC and PE are hypothesized to contribute to intestinal epithelial membrane stability, potentially helping the fish adjust to changes in dietary lipid composition [47,48]. Second, the upregulation of *dgki* promotes the conversion of PA to diacylglycerol (DG), which, together with DG accumulation resulting from the downregulation of *dgke* and *dgkh*, may drive triacylglycerol (TG) synthesis and accumulation. This metabolic pattern, characterized by decreased degradation products (e.g., LPC, FFA, PA) and increased synthetic lipids (e.g., PC, PE, TG), suggests a shift toward lipid storage rather than oxidation [49]. Third, changes in arachidonic acid metabolism-related genes and metabolites (e.g., *alox15b*, *gpx4*, 5-HETE, 15-HETE) are associated with a low-inflammatory state, as suggested by reduced levels of pro-inflammatory mediators [50]. Additionally, epoxy fatty acids (9,10-EpOME and 12,13-EpOME), which accumulated during domestication, are known activators of PPARγ and Nrf2 pathways in other species [51,52], and their increase might enhance fatty acid transport efficiency and support antioxidant defense. Concurrent upregulation of *pla2g4c* facilitated phospholipid absorption, inhibited fatty acid oxidation, and further supported intestinal homeostasis [53]. However, these proposed mechanisms are based on correlative evidence and require further experimental validation in future studies.

Despite the integrative approach and the insights gained from this study, several limitations should be acknowledged. As discussed above, the S3′ control group and the multi-omics filtering strategy allowed us to partially disentangle developmental and dietary effects. However, the domestication process still involves concurrent changes in age and diet, and a fully crossed design would provide stronger separation of these two factors. Additionally, while our multi-omics analysis revealed significant enrichment of lipid metabolism pathways and proposed potential molecular mechanisms associated with feed adaptation, these findings are based on correlative evidence. Functional validation is required to establish causal relationships, such as inhibitor assays, gene knockouts and targeted metabolomics. Furthermore, all fish were obtained from a single commercial source and reared in ponds at one location, which may limit the generalizability of our findings to other populations or farming conditions. Moreover, this study followed fish only up to 1-year-old; long-term (>1 year) performance, including reproductive success and multi-cycle growth, remains unknown. Future studies addressing these limitations will further strengthen the evidence base for feed domestication in *C. nasus*.

## 5. Conclusions

*C. nasus* is a highly valuable migratory species, and artificial cultivation has become an important approach for the conservation and utilization of its rare germplasm resources. However, heavy reliance on natural bait and limited adaptation to formulated feed significantly constrain the development of its aquaculture industry. In this study, we found that during the domestication from natural bait to formulated diet, *C. nasus* exhibited marked structural remodeling of digestive tissues, significant increases in intestinal digestive enzyme activities, and enhanced gut microbial diversity, particularly a notable rise in Firmicutes abundance, although the natural bait group (S3′) still exhibited higher Firmicutes abundance than the formulated-feed group (S3). These findings suggest that feed adaptation in *C. nasus* is accompanied by substantial changes in digestive capacity and microbial ecology. Multi-omics analysis revealed significant enrichment of lipid metabolism-related pathways. Based on correlative data, we propose a hypothetical model in which differentially expressed genes (e.g., *lpl*, *chpt1*, *gpx4*, *pld1*, *dgki*) and metabolites (e.g., phosphatidylcholine, phosphatidylethanolamine, triacylglycerol) may be associated with the adaptive response to formulated feed, including alterations in membrane stability, lipid metabolism, and inflammatory state. Importantly, no significant growth differences were observed between fish fed a formulated diet and those maintained on natural bait, confirming the feasibility of feed replacement from a practical perspective. Overall, the findings confirm the feasibility of artificial domestication and elucidate the molecular response networks underlying dietary adaptation, providing scientific evidence for replacing natural bait with formulated diets in *C. nasus* aquaculture.

## Figures and Tables

**Table 1:** Quantitative primer information.

Primer Name	Forward Primer Sequence (5′-3′)	Reverse Primer Sequence (5′-3′)	Product Length (bp)
*lpl*	TGGAGTGGCAGGAAACCTCA	CAGGCCTCTGGATGCCAATG	185
*chpt1*	GCTGTGGGATCGGGAGGTAT	GGAAGGCGTACAGCTTCAGG	234
*gpx4*	GGTTTACGCATCCTGGCCTT	TTCCGAAGAGTCCCTTGCCA	196
*cyp3a27*	CTGTTCGCGGGCTATGAGAC	GAGCAGCAGGAGGGTACAGA	208
*ept1*	TCGACTTCTACGCCTCAGCA	GCCATCCACTCCGTCCAATG	107
*pld1*	CCACAGGAGGTGGAAATGCC	GTCGTCGGCAATGAGCATCT	218
*pnpla4*	AGTGGCCCTCTGGACATCTG	AAGAGCGCCTGGTTTAGACG	119
*mogat2a*	GCCAACAAGAAGGGCTTTATC	CCTCCATGTAGAGCTGGTGAA	122
*β-actin*	CCAGGCATCAGGGTATGGGA	CGCAGCTCGTTGTAGAAGGT	160

**Table 2:** Diversity indices of gut microbiota of *C. nasus* at different stages.

Group	Observed Amplicon Sequence Variants	Goods Amplicon Sequence Variants
S1	376	0.999
S2	604	0.998
S3	511	0.998
S3′	210	0.998

## Data Availability

The original data presented in the study are included in the article/Supplementary Materials. Further inquiries can be directed to the corresponding authors.

## Supplementary Materials

The following supporting information can be downloaded at: https://www.mdpi.com/article/10.3390/ani16101518/s1, Table S1: Nutrient indexes of the natural bait and the formulated diet; Table S2: qPCR Primer Design and Evaluation of Amplification Efficiency; Table S3: Evaluation of nutrient composition of *C. nasus* at different stages; Table S4: Differential metabolites of *C. nasus* identified by intestinal metabolome with potential involvement in the feed domestication process; Table S5: Differentially expressed genes of *C. nasus* identified by intestinal transcriptome with potential involvement in the feed domestication process.

## Abbreviations

The following abbreviations are used in this manuscript:

H	Hepatocyte
V	Vacuole
GGCs	Gastric gland cells
GPs	Gastric pits
CCs	Columnar cells
IV	Intestinal villus
FDs	Fat droplets
CML	Circular muscle layer
VGCs	Vacuolated goblet cells
ML	Muscular layer of stomach wall
PC	Phosphatidylcholine
LPC	Lysophosphatidylcholine
PE	Phosphatidylethanolamine
MG	Monoglyceride
LPA	Lysophosphatidic acid
PA	Phosphatidic acid
FFA	Free fatty acid
5-HETE	5-hydroxyeicosatetraenoic acid
15-oxoETE	15-oxo eicosatetraenoic acid
(±)15-HETE	±15-hydroxyeicosapentaenoic acid
PS	Phosphatidylserine
TG	Triglyceride
